# Supplementation of dietary semen vaccariae extracts to lactating sow diets: effects on the production performance, milk components, and gene expression related to mammogenesis

**DOI:** 10.3389/fvets.2023.1284552

**Published:** 2023-11-09

**Authors:** Chaohua Xu, Jiajun Xie, Fengjie Ji, Weiqi Peng, Yuzhuo Song, Xinping Diao, Hongzhi Wu

**Affiliations:** ^1^Tropical Crop Genetic Resource Research Institute, Chinese Academy of Tropical Agricultural Sciences, Haikou, China; ^2^College of Animal Science and Technology, Northeast Agricultural University, Harbin, China; ^3^Zhanjiang Experimental Station, Chinese Academy of Tropical Agricultural Sciences, Zhanjiang, China; ^4^Department of Animal Husbandry and Veterinary Medicine, Shijiazhuang Information Engineering Vocational College, Shijiazhuang, China

**Keywords:** semen vaccariae extracts, lactating sows, production performance, colostrum components, mammogenesis

## Abstract

This study aimed to investigate the effects of dietary semen vaccariae extracts (SVE) on the production performance, colostrum components, and relative gene expression related to mammogenesis of lactating sows. 48 pregnant sows were selected and randomly allocated into four groups, with six replicates and two sows per replicate. The first group was the control (CON), while the other groups received the same diet further supplemented with 1.5, 3.0 and 4.5 g SVE per kg (SV1, SV2 and SV3, respectively). Compared with the control group, (1) the average daily gain was increased (*p* < 0.05) in SV1, SV2, and SV3 during the 11–21 days and 1–21 days of lactation; (2) the serum insulin-like growth factor-1, insulin, prolactin, and estrogen contents in SV1, SV2, and SV3 were increased (*p* < 0.05) on the 1st and 21st day of lactation; (3) The plasma Lysine, Threonine, and Tryptophan concentrations were also higher (*p* < 0.05) in SV1, SV2, and SV3 on the 1st and 21st day of lactation; (4) The milk Lysine, Methionine, Threonine, and Tryptophan concentrations were higher (*p* < 0.05) in SV1, SV2, and SV3 on the 1st and 21st day of lactation; (5) The milk lactose ratio and milk protein content were increased (*p* < 0.05) in the groups treated with semen vaccariae on the 1st day of lactation, while the milkfat ratio and milk protein content were increased (*p* < 0.05) in SV2 and SV3 on the 21st day of lactation; (6) the immunoglobulin M, A, and G contents were increased (*p* < 0.05) in the groups treated with the semen vaccariae on the first day of lactation; and (7) the relative *PRLR*, *STAT5a*, *FcRn*, *CSN2*, and *LALBA* expressions were higher (*p* < 0.05) in the groups treated with the semen vaccariae on the 1st and 21st day of lactation. In this study, the optimum dosage was 3.0 g/kg semen vaccariae, which increased the average daily gain of piglets, total lactation yield, and serum hormone levels, improved the amino acid levels in plasma, and facilitated the milk quality, up-regulated the relative gene expressions in the mammogenesis.

## 1. Introduction

The mammary gland is a complex secretory organ composed of many different types of cells, including vascular endothelial cells, fibroblasts, immune cells, and epithelial cells, unique to mammals (1, 2). During gestation, the mammary gland undergoes extensive tissue remodeling under hormonal regulation, including ductal branche proliferation, acinar bud development, and extracellular matrix remodeling (3). At the early stages of gestation, the mammary ductal epithelium proliferates and occupies the intraductal space, interstitial adipose tissue gradually disappears, and angiogenesis increases; at the middle stages of gestation, the developing ductal branches are surrounded by a network of capillaries; and at the late stages of gestation, as the mammary glands differentiate, alveoli reach their development peak, which covers the majority of the fat pad and exhibits some secretory activity as gestation approaches (4–6). During lactation, the mammary epithelial cells act as a double-layered tubular structure, discharging milk from the acinar lumen and supplying the young animals with immunoglobulins and energy, thus decreasing intestinal diseases and sustaining the survival and growth of the young animals (7). The gradual decrease in demand for milk as young animals grow and develop leads to stagnation of milk in the acinar lumen, which initiates the degenerative process, i.e., of epithelial cell apoptosis and the epithelial ductal tree remodeling (8, 9). The colostrum is crucial for the survival of newborn piglets, as the immunoglobulin of sow cannot pass the placental barrier (10, 11). The newborn piglets can only get the maternal immunoglobulin through colostrum, and all the early nutrients of piglets come from breast milk (12–14), so improving the lactation yield of sows to make piglets grow fast and healthy is also the top priority in pig production.

Semen vaccariae, the seed of *Vaccaria segetalis*, help improving the symptoms of women’s postpartum milk shortage and is traditionally used in East Asian countries for breast milk deficiency treatment because of its low price and good efficacy (15). However, its use in local breed sows and its effect on mammogenesis in sows have not yet been reported. We hypothesized that semen vaccariae also has the effect of promoting milk secretion and mammogenesis in gestating and lactating sows, so the objective of this study was to (a) examine the effects of semen vaccariae extracts on the production performance, milk components, and gene expression related to mammogenesis of lactating sows; (b) preliminary investigate the action mechanism of semen vaccariae on the lactation ability of sows; and (c) examine the valuable addition of semen vaccariae extracts in the feed of gestating and lactating sows.

## 2. Materials and methods

### 2.1. Experimental material

Forty eight primiparous sows of the Hainan local breed (Tunchang), with an average body weight of 100.00 ± 8.00 kg, the same genetic background and similar birth date were used.

Semen vaccariae extracts (SVE) were purchased from Shaanxi Hao Yang Biotechnology Co. The SVE active ingredient contents were determined by the liquid chromatograph and mass spectrometer in the Pony Testing International Group Company, Beijing, China. The semen vaccariae flavonoids content of SVE was 4.82%, the saponin content was 1.73%, and the vacsegoside content was 0.62%, detected by 1,260 Infinity II High-Performance Liquid Chromatograph, Agilent, Beijing, China.

Serum biochemical and colostrum immunoglobulin kits were purchased from Shanghai Sangon Biotechnology Co., Ltd., Shanghai, China.

### 2.2. Experiment design and sample collection

The experiment adopted a single-factor design. Forty-eight 317-day-old primiparous sows in the third semester of pregnancy (107 days), with similar physical conditions and expected confinement period, were selected and were randomly allocated into four treatments with six replicates and two sows per replicate. The basal diets for the gestating and lactating sows were a corn-soybean meal type diet formulated according to the NRC (1998; 2012) standards for swine feeding, and the composition of the basal diets and nutritional levels are shown in Table 1. The first group was the control (CON), while the other groups received the same diet further supplemented with 1.5, 3.0 and 4.5 g SVE per kg (SV1, SV2 and SV3, respectively). Each group was fed the corresponding experimental feed from the 107th day of sow gestation, restricted to 3.0 kg per day for 3 days before the farrowing date. Every sow was fed 2.0 kg on the first day after farrowing and increased by 0.5–1.0 kg per day after that. The sow had then free access to feed and water. The piglet number was adjusted to 9–11 per lactating sow within 3 days after farrowing by cross-fostering. In the farrowing room, piglets were kept warm with thermal insulation and warming lamps.

**Table 1:** Composition (kg/100 kg) of the basal experimental diets^a^ for gestating and lactating sows.

Ingredients	Content	Nutrient levels	Content
Corn, %	69.00	Digestible energy^c^, DE, MJ/kg	13.98
Wheat bran, %	3.00	Crude protein^d^, CP, %	16.35
Soybean meal, %	19.00	Calcium^d^, Ca, %	0.73
Fish meal, %	2.00	Available phosphorus^d^, AP, %	0.34
Soybean oil, %	2.60	Lysine^d^, Lys, %	0.92
CaHPO_4_, %	0.70	Methionine^d^, Met, %	0.26
Stone powder, %	1.00	Threonine^d^, Thr, %	0.59
NaCl, %	0.70		
Premix^b^, %	2.00		
Total	100		

a Based on the NRC (1998; 2012) nutrient requirements for lactating sows.

b The premix provided the following per kg of diet: VA 2000 IU, VD 200 IU, VE 45 IU, VK 0.5 mg, VB_1_ 1 mg, pantothenic acid 12 mg, nicotinic acid 10.25 mg, VB_6_ 3.85 mg, VB_12_ 15 μg, folic acid 1.35 mg, biotin 0.21 mg, Mn as manganese sulfate 20 mg, Fe as ferrous sulfate 80 mg, Cu as copper sulfate 5 mg, I as potassium iodide 0.14 mg, and Se as sodium selenite 0.15 mg.

c Calculated value (NRC, 1998; 2012).

d Analysed content.

The experiment lasted from the 107th day of gestation to 10 days after the weaning of piglets on the 21st day. The number of piglets and the weight of piglets per sow on the 1st and 21st days were recorded to calculate the average daily weight gain (ADG) of piglets at different stages or throughout the feeding period. In addition, the average daily feed intake (ADFI) of the lactating sows and the number of piglet-weaning litters (NWL) were recorded throughout the feeding period. The sows were observed in estrus interval (EI) from the fourth day after weaning, and the time between estrus was recorded. If the EI was more than 10 days, it was recorded as no estrus. The total lactation yield (TLY) during lactation was calculated using the formula (16, 17):


$$\begin{array}{l} \mathrm{Total}\;\mathrm{lactation}\;\mathrm{yield},\mathrm{kg}=\mathrm{Average}\;\mathrm{daily}\;\mathrm{weight}\;\mathrm{gain}\;\mathrm{of}\;\mathrm{piglets}\\ \times \mathrm{Number}\;\mathrm{of}\;\mathrm{lactation}\;\mathrm{days}\times \mathrm{Number}\;\mathrm{of}\;\mathrm{litters}\times 4. \end{array}$$

About 30 mL of colostrum were collected with pulsating vacuum pump for sow milking (ZL001), shanghai, China, from the front, middle, and rear nipples of the sows within 2 h after the end of parturition and on the 21st day of lactation, then mixed well and stored at −20°C until further testing. On the 107th day of gestation, 1st and 21st days of lactation, 10 mL of blood was collected with disposable vacuum blood collection tubes from the ear vein of each sow. After resting the blood for 15 min, it was centrifuged at 3000 rpm for 15 min to obtain the serum with Multifuge X4 Pro ThermoFisher High performance centrifuge, Shanghai, China, which was future transferred into Eppendorf tubes and stored at −20°C for testing. The milk protein content (MPC), milk fat ratio (MFR), milk lactose ratio (MLR), and solids-not-fat (SNF) in milk were determined using a near-infrared reflectance spectroscopy instrument (Milk-Scan 134 A/B, Beijing, China). Prior to analysis, colostrum and milk were separated by centrifugation at 3000 × g at 4°C for 20 min. In addition, the Insulin-like growth factor-1 (IGF-1), Insulin (INS), prolactin (PRL), and estrogen (E) in serum, and the Immunoglobulin A (IgA), Immunoglobulin G (IgG), Immunoglobulin M (IgM) in colostrum were determined by the HITACHI Automatic Analyzer 3,500, Ibaraki-Ken, Japan. The assay was performed according to the kit instructions. The variation coefficients of all indexes in inter-and intra-assay were controlled within 5.00%. About 5 mL of blood (plasma) and milk were collected with disposable vacuum blood collection tubes and stored at −20°C for amino acids test by 1,260 Infinity II High-Performance Liquid Chromatograph, Agilent, Beijing, China.

On the 1st and 21st day of lactation, 4 mL lidocaine hydrochloride (2 mL:40 mg, Beijing Yimin Pharmaceutical Co., Ltd., Beijing, China) was injected into the second left papilla of the sows for local anesthesia, and 3 g of mammary tissue samples were collected using a live sampling gun (BARD^®^ MAGNUM^®^, MG1522, United States) and disposable sampling needles (C.R. Bard. Inc., Coington GA, United States). The total RNA of the mammary tissue was isolated with TRIzol (Sigma, Saint Louis, MO) according to the RNA extraction method. The total RNA from each mammary tissue was reverse transcribed into cDNA using a Prime Script™ RT reagent kit with gDNA Eraser (TaKaRa, Dalian, China). The obtained cDNA was used for qPCR using a TB Green™ Premix Ex Taq™ PCR kit (TaKaRa, Dalian, China). The primer sequences are shown in Table 2, and the primers corresponding to the pig gene sequence were synthesized by Sangon (Shanghai, China). Samples were disposed of with an ABI PRISM 7500 SDS thermal cycler (Applied Biosystems, Foster City, CA, United States), and the following temperature program was used: one cycle at 95°C for 30 s, 40 cycles of 95°C for 5 s, and 60°C for 34 s. Based on the 2^−∆∆Ct^ method, relative gene mRNA expression was the mean of the normalized by β-actin and Glyceraldehyde-3-phosphate dehydrogenase expression, respectively. Moreover, all of the processes were performed in triplicates under RNase-free conditions. RT-qPCR products were cloned into a pMD18-T vector (TaKaRa) and sequenced by the Sanger method. The sequencing results were compared with the gene sequences in NCBI, and the genes amplified by the primers in Table 2 were verified as the target genes according to the alignment sequence results (16).

**Table 2:** Primer Sequence list.

Gene	Gene name	Forward and reverse primers	Product size	Accession No.
PRLP	Prolactin receptor	F:5’-GGCTCCGTTTGAAGAACCAA-3’	67	NM_001001868.1
		R:5’-GTCTTTCGCAGCTGGATTCTG-3’		
LALBA	α-Lactalbumin	F: 5’-GGATTGACTACTGGTTGGCCC-3’	100	NM_214360.1
		R: 5’-GGCAGAAGCAGCAAGACAGC-3’		
CSN2	β-Casein	F: 5’-GGACTTGATCGCCATGAAGCT-3’	81	NM_214434.2
		R: 5’-GCATTGAGTTCTTCCTTCGCTCT-3’		
FCRn (PCRGT)	Neonatal Fc receptor	F: 5’-TTTGGGCCTGACTTTTGTGT-3’	61	NM_214197.2
		R: 5’-AATTCCCCCCACGTTGC-3’		
STAT5b	Signal transduction and activator of transcription 5b	F: 5’-GACTCTGAAATTGGTGGCATCA-3’	67	NM_214168.1
		R: 5’-GATTCCAAAACATTCTTTCCTCAGA-3’		
STAT5a	Signal transduction and activator of transcription 5a	F: 5’-ATCTCATCTATGTGTTTCCCGACC-3’	74	NM_214190.1
		R: 5’-CGGAGCGAGCACAGGAGT-3’		
GAPDH	Glyceraldehyde-3-phosphate dehydrogenase	F: 5’-GTCGGAGTGAACGGATTTGG-3’	76	NM_001206359.1
		R: 5’-CAATGTCCACTTTGCCAGAGTTAA-3’		
β-actin	–	F: 5’-AGGCTACAGCTTCACCACCAC-3’	95	AB618546
		R: 5’-CCATCTCCTGCTCAAAATCCA-3’		

### 2.3. Statistical analysis

Statistical analyses were conducted using SPSS statistics software (version 20.0, International Business Machines Corporation, Armonk, NY, United States). Data were expressed as mean ± SEM. Statistical comparisons of different treatments were performed using one-way ANOVA or Welch ANOVA after the variance homogeneity test. Statistical differences among groups were assessed using Duncan’s multiple range test with dietary treatment as a fixed factor. Each replicate was considered as an experimental unit. The test results of all analyses were considered significant at *p* < 0.05.

## 3. Results

### 3.1. Semen vaccariae extracts improved the production performance of lactating sows

Compared with the control group, the ADG in SV2 and SV3 was increased (*p* < 0.05) by 10.00 and 9.50%, respectively, during the first 10 days of lactation, and it was increased (*p* < 0.05) by 4.15% vs. 6.86, 7.83% vs. 11.27, 8.29% vs. 11.27%, respectively, in SV1, SV2, and SV3 during the 11–21 days and 1–21 days of lactation. The TLY was 7.02, 11.70, and 11.70%, respectively, higher (*p* < 0.05) in the groups treated with the senmen vaccariae extracts than that in the control group, and it was 11.70% vs. 11.70% higher (*p* < 0.05) in SV2 and SV3 than that in SV1 (Table 3).

**Table 3:** Effect of semen vaccariae extracts on the production performance of lactating sows.

Parameters	Con	SV1	SV2	SV3	*p*-value
ADG, g/d					
1–10 d	200±6.82^b^	210±3.39^ab^	220±5.64^a^	219±5.23^a^	0.0431
11–21 d	217±4.26^c^	226±3.95^b^	234±3.53^a^	235±4.36^a^	0.0236
1–21 d	204±5.21^c^	218±3.29^b^	227±3.24^a^	227±4.03^a^	0.0328
ADFI, kg/d	5.64±0.26	5.68±0.32	5.69±0.37	5.71±0.45	0.0526
TLY, kg	171±5.21^c^	183±3.61^b^	191±3.59^a^	191±4.22^a^	0.0356
EI, d	6.01±0.07	6.00±0.10	5.99±0.09	5.98±0.08	0.1584
NWL	9.98±0.45	10.01±0.26	10.23±0.15	10.22±0.23	0.0646

Values with different small letter superscripts in the same column mean a significant difference (*p* < 0.05). ADG, average daily gain of piglets; ADFI, average daily feed intake of lactating sows; EI, estrus interval; TLY, total lactation yield; NWL, number of piglet weaning litters.

### 3.2. Semen vaccariae extracts improved the serum hormone levels of lactating sows

There were no significant differences (*p* > 0.05) in the serum IGF-1, INS, PRL, and E contents on the 107th day of gestation. The serum IGF-1, INS, PRL and E contents were 11.25, 12.50 and 13.13% vs. 9.12, 9.41 and 9.29% vs. 6.35, 12.46 and 12.14% vs. 11.06, 18.36 and 10.93%, respectively, higher (*p* < 0.05) in the groups treated with the semen vaccariae than those in the control group on the first day of lactation. The serum IGF-1, INS, PRL, and E contents were 7.62, 13.81 and 8.57% vs. 32.86, 43.68 and 50.05% vs. 8.68, 10.95 and 11.03% vs. 9.40, 10.94 and 12.38%, respectively, higher (*p* < 0.05) in the groups treated with the semen vaccariae than those in the control group, the IGF-1 contents in SV2 were 5.75% vs. 4.82% higher (*p* < 0.05) than those in SV1and SV3, and the INS contents were increased (*p* < 0.05) 8.15% vs. 12.94% in SV2 and SV3 compared with SV1 on the 21st day of lactation (Table 4).

**Table 4:** Effect of semen vaccariae extracts on the serum hormone levels of lactating sows.

Parameters	Con	SV1	SV2	SV3	*p*-value
107th day of gestation					
IGF-1, ng/mL	136±7.33	139±9.16	138±6.98	137±9.26	0.5672
INS, mIU/L	11.12±0.75	12.30±1.26	11.59±0.96	11.63±1.22	0.6829
PRL, ng/mL	64.02±0.89	65.31±0.95	64.93±0.89	64.86±0.67	0.7596
E, pg/mL	30.06±0.43	31.26±0.59	30.64±0.98	30.43±0.94	0.6524
1st day of lactation					
IGF-1, ng/mL	160±9.23^c^	178±5.62^b^	180±6.55^a^	181±8.36^a^	0.0462
INS, mIU/L	17.65±0.75^c^	19.26±0.53^b^	19.31±0.98^a^	19.29±0.82^a^	0.0395
PRL, ng/mL	80.26±0.95^c^	85.36±0.81^b^	90.26±0.76^a^	90.00±0.98^a^	0.0403
E, pg/mL	54.26±0.89^c^	60.26±0.98^b^	64.22±0.58^a^	60.19±0.99^b^	0.0443
21st day of lactation					
IGF-1, ng/mL	210±9.36^c^	226±5.91^b^	239±5.24^a^	228±4.36^b^	0.0429
INS, mIU/L	21.06±0.95^c^	27.98±0.86^b^	30.26±0.64^a^	31.60±0.68^a^	0.0356
PRL, ng/mL	70.26±0.95^b^	76.36±0.65^a^	77.95±0.87^a^	78.01±0.68^a^	0.0356
E, pg/mL	46.27±0.95^b^	50.62±0.86^a^	51.33±0.62^a^	52.00±0.95^a^	0.0452

Values with different small letter superscripts in the same column mean a significant difference (*p* < 0.05). IGF-1, insulin-like growth factor-1; INS, insulin; PRL, prolactin; E, estrogen.

### 3.3. Semen vaccariae extracts improved the plasma-free amino acid concentration of lactating sows

The plasma Lys, Thr, and Try concentrations were 17.47, 38.18 and 38.70% vs. 10.49, 14.10 and 13.78% vs. 23.27, 54.88 and 24.33%, respectively, higher (*p* < 0.05) in the groups treated with semen vaccariae than those in the control group, and they were increased (*p* < 0.05) by 17.63 and 18.08% vs. 3.27 and 2.98% vs. 25.65 and 0.86%, respectively, in SV2 and SV3 compared with SV1 on the first day of lactation. The plasma Lys, Met, Thr, and Try concentrations were 31.56, 56.66 and 56.95% vs. 23.08, 39.40 and 39.02% vs. 12.84, 26.39 and 13.01% vs. 40.50, 90.23 and 42.10%, respectively, higher (*p* < 0.05) in the groups treated with semen vaccariae than those in the control group on the 21st day of lactation. The plasma Lys and Met concentrations in SV2 and SV3 were increased (*p* < 0.05) by 19.08 and 19.30% vs. 13.26 and 12.96%, respectively, compared with SV1, and the plasma Thr and Try concentrations in SV2 were 12.01% vs. 11.84 and 35.40% vs. 33.87%, respectively, higher (*p* < 0.05) than those in SV1 and SV3 on the 21st day of lactation (Table 5).

**Table 5:** Effect of semen vaccariae extracts on the plasma-free amino acid concentration of lactating sows.

Parameters	Con	SV1	SV2	SV3	*p*-value
1st day of lactation					
Valine (Val), μg/mL	23.60±0.89	24.63±0.92	24.00±0.86	23.98±0.95	0.5674
Leucine (Leu), μg/mL	15.23±0.63	15.32±0.89	15.33±0.68	15.43±0.98	0.6259
Isoleucine (Ile), μg/mL	7.23±0.56	7.33±0.98	7.35±0.54	7.36±0.76	0.5561
Lysine (Lys), μg/mL	9.56±0.26^c^	11.23±0.63^b^	13.21±0.29^a^	13.26±0.41^a^	0.0456
Methionine (Met), μg/mL	6.32±0.25	6.36±0.39	6.43±0.98	6.41±0.76	0.3896
Cysteine (Cys), μg/mL	3.65±0.26	3.70±0.62	3.71±0.81	3.70±0.58	0.1254
Phenylalanine (Phe), μg/mL	13.65±0.95	13.70±0.86	13.71±0.65	13.70±0.86	0.3276
Threonine (Thr), μg/mL	31.28±1.31^c^	34.56±2.35^b^	35.69±3.24^a^	35.59±3.69^a^	0.0472
Glycine (Glu), μg/mL	29.11±1.20	29.23±1.89	30.24±3.12	30.20±3.56	0.0675
Arginine (Arg), μg/mL	21.87±2.21	22.36±3.51	22.52±3.87	21.98±3.21	0.1542
Alanine (Ala), μg/mL	64.25±3.56	65.21±4.25	64.29±5.62	65.22±5.82	0.2681
Serine, Ser, μg/mL	9.71±0.23	9.72±0.62	9.87±0.33	9.85±0.43	0.1583
Glycine (Gly), μg/mL	30.56±3.12	31.26±3.56	31.32±3.81	31.40±3.82	0.2634
Histidine (His), μg/mL	10.26±0.95	10.36±0.98	10.29±0.99	10.39±0.98	0.1587
Tryptophan (Try), μg/mL	5.63±0.98^c^	6.94±0.89^b^	8.72±0.86^a^	7.00±0.69^b^	0.0421
21st day of lactation					
Valine (Val), μg/mL	19.60±0.89	20.36±0.92	20.00±0.75	20.98±0.97	0.4755
Leucine (Leu), μg/mL	13.33±0.67	13.36±0.79	13.37±0.58	13.41±0.72	0.2653
Isoleucine (Ile), μg/mL	5.24±0.66	5.36±0.78	5.37±0.94	5.36±0.97	0.6532
Lysine (Lys), μg/mL	10.36±0.36^c^	13.63±0.67^b^	16.23±0.46^a^	16.26±0.98^a^	0.0306
Methionine (Met), μg/mL	5.33±0.37^c^	6.56±0.35^b^	7.43±0.21^a^	7.41±0.29^a^	0.0396
Cysteine (Cys), μg/mL	4.62±0.36	4.71±0.92	4.70±0.93	4.70±0.85	0.2652
Phenylalanine (Phe), μg/mL	10.56±0.55	10.70±0.96	10.70±0.94	10.70±0.99	0.4320
Threonine (Thr), μg/mL	29.82±1.25^c^	33.65±1.24^b^	37.69±2.36^a^	33.70±2.62^b^	0.0332
Glycine (Glu), μg/mL	26.51±1.23	26.23±1.99	28.22±3.15	28.20±3.96	0.1267
Arginine (Arg), μg/mL	20.89±2.29	21.33±3.49	21.52±3.93	21.42±3.37	0.1643
Alanine (Ala), μg/mL	60.27±3.57	60.25±4.22	60.30±5.21	60.29±5.96	0.2762
Serine (Ser), μg/mL	6.72±0.24	6.72±0.42	6.87±0.63	6.85±0.73	0.2586
Glycine (Gly), μg/mL	37.55±3.62	37.26±3.96	37.32±3.91	37.40±3.89	0.3631
Histidine (His), μg/mL	9.21±0.93	9.31±0.99	9.29±0.89	9.39±0.88	0.1080
Tryptophan (Try), μg/mL	5.63±0.78^c^	7.91±0.76^b^	10.71±0.86^a^	8.00±0.76^b^	0.0453

Values with different small letter superscripts in the same column mean a significant difference (*p* < 0.05).

### 3.4. Semen vaccariae extracts improved the milk-free amino acid concentration of lactating sows

The milk Lys, Met, Thr, and Try concentrations were 65.86, 117.82 and 66.47% vs. 36.36, 69.70 and 90.91% vs. 13.51, 16.22 and 14.41% vs. 26.79, 44.64 and 48.21%, respectively, higher (*p* < 0.05) in the groups treated with semen vaccariae than those in the control group; the milk Lys concentration in SV2 was increased (*p* < 0.05) by 31.33% vs. 30.85%, respectively, compared with SV1 and SV3, the milk Met concentration in SV3 was increased (*p* < 0.05) by 40.00% vs. 12.50%, respectively, compared with SV1 and SV2, the milk Try concentrations in SV2 and SV3 were increased (*p* < 0.05) by 14.08% vs. 16.90%, respectively, compared with SV1 on the first day of lactation. The milk Lys, Met, Thr, and Try concentrations were 10.46, 33.39 and 33.48% vs. 51.46, 86.41 and 87.38% vs. 12.14, 24.64 and 33.93% vs. 28.57, 59.52 and 66.67%, respectively, higher (*p* < 0.05) in the groups treated with semen vaccariae than those in the control group; the milk Lys, Met, and Try concentrations were increased (*p* < 0.05) by 13.27, 23.08 and 24.07% vs. 13.35, 23.72 and 29.63%, respectively, in SV2 and SV3 compared with SV1, the milk Thr concentration in SV3 was 19.43% vs. 7.45%, respectively, higher (*p* < 0.05) than that in SV1 and SV2 on the 21st day of lactation (Table 6).

**Table 6:** Effect of semen vaccariae extracts on the milk-free amino acid concentration of lactating sows.

Parameters	Con	SV1	SV2	SV3	*p*-value
1st day of lactation					
Valine, Val, μg/mL	1.13±0.08	1.12±0.05	1.13±0.08	1.13±0.06	0.1265
Leucine, Leu, μg/mL	1.52±0.02	1.51±0.03	1.52±0.04	1.52±0.06	0.2634
Isoleucine, Ile, μg/mL	0.89±0.07	0.90±0.09	0.91±0.08	0.90±0.07	0.8237
Lysine, Lys, μg/mL	3.31±0.09^c^	5.49±0.06^b^	7.21±0.09^a^	5.51±0.09^b^	0.0345
Methionine, Met, μg/mL	0.33±0.02^d^	0.45±0.04^c^	0.56±0.02^b^	0.63±0.03^a^	0.0202
Cysteine, Cys, μg/mL	2.21±0.07	2.23±0.09	2.21±0.06	2.21±0.08	0.5621
Phenylalanine, Phe, μg/mL	1.68±0.09	1.68±0.06	1.65±0.09	1.68±0.07	0.6892
Threonine, Thr, μg/mL	1.11±0.05^b^	1.26±0.04^a^	1.29±0.05^a^	1.27±0.08^a^	0.0436
Glycine, Glu, μg/mL	3.82±0.16	3.83±0.26	3.79±0.65	3.82±0.51	0.5281
Arginine, Arg, μg/mL	2.18±0.15	2.16±0.20	2.19±0.19	2.21±0.23	0.6257
Alanine, Ala, μg/mL	3.95±0.30	3.96±0.42	4.00±0.52	4.01±0.32	0.7612
Serine, Ser, μg/mL	3.12±0.33	3.13±0.29	3.10±0.39	3.12±0.42	0.8618
Glycine, Gly, μg/mL	3.86±0.17	3.89±0.26	3.91±0.42	3.90±0.40	0.6213
Histidine, His, μg/mL	0.75±0.01	0.74±0.02	0.75±0.03	0.76±0.03	0.4525
Tryptophan, Try, μg/mL	0.56±0.01^c^	0.71±0.03^b^	0.81±0.06^a^	0.83±0.07^a^	0.0362
21st day of lactation					
Valine, Val, μg/mL	2.01±0.02	2.02±0.02	2.02±0.04	2.04±0.03	0.5628
Leucine, Leu, μg/mL	2.49±0.12	2.50±0.21	2.51±0.11	2.50±0.19	0.6215
Isoleucine, Ile, μg/mL	0.55±0.02	0.52±0.03	0.53±0.04	0.55±0.04	0.4283
Lysine, Lys, μg/mL	11.26±0.22^c^	13.26±0.34^b^	15.02±0.35^a^	15.03±0.62^a^	0.0473
Methionine, Met, μg/mL	1.03±0.01^c^	1.56±0.02^b^	1.92±0.04^a^	1.93±0.03^a^	0.0328
Cysteine, Cys, μg/mL	3.94±0.06	3.93±0.07	3.96±0.09	3.97±0.09	0.5621
Phenylalanine, Phe, μg/mL	9.26±0.89	9.27±0.62	9.31±0.98	9.30±0.91	0.6587
Threonine, Thr, μg/mL	11.20±0.12^d^	12.56±0.32^c^	13.96±0.53^b^	15.00±0.92^a^	0.0437
Glycine, Glu, μg/mL	60.12±1.26	61.02±2.36	61.07±2.61	61.23±3.29	0.0835
Arginine, Arg, μg/mL	10.96±0.12	10.89±0.23	10.92±0.63	1.86±0.52	0.8624
Alanine, Ala, μg/mL	24.45±1.23	24.56±1.96	24.90±2.65	24.89±3.21	0.9561
Serine, Ser, μg/mL	5.46±0.26	5.36±0.62	5.46±0.86	5.64±0.95	0.8624
Glycine, Gly, μg/mL	21.82±3.55	21.86±4.02	21.89±3.92	21.87±4.23	0.9887
Histidine, His, μg/mL	3.76±0.42	3.75±0.46	3.78±0.52	3.80±0.62	0.5612
Tryptophan, Try, μg/mL	0.42±0.01^c^	0.54±0.03^b^	0.67±0.05^a^	0.70±0.03^a^	0.0406

Values with different small letter superscripts in the same column mean a significant difference (*p* < 0.05).

### 3.5. Semen vaccariae extracts improved the milk composition of lactating sows

The MLR and MPC contents were increased (*p* < 0.05) by 39.91, 72.77 and 88.26% vs. 23.89, 40.85 and 40.94%, respectively, in the groups treated with semen vaccariae compared with the control group. The MPC contents were 13.69% vs. 13.76%, respectively, higher (*p* < 0.05) in SV2 and SV3 than those in SV1, the MLR contents in SV3 were 34.56% vs. 8.96%, respectively, higher (*p* < 0.05) than those in SV1 and SV2, the MFR contents were increased (*p* < 0.05) by 54.97% vs. 55.53%, respectively, in SV2 and SV3 compared with the control group on the first day of lactation. The MFR and MPC contents were increased (*p* < 0.05) by 83.41 and 84.13% vs. 30.12 and 35.96% in SV2 and SV3 compared with the control group; the MLR contents were 25.49, 47.06 and 48.04%, respectively, higher (*p* < 0.05) than those in the control group on the 21st day of lactation (Table 7).

**Table 7:** Effect of semen vaccariae extracts on the milk composition of lactating sows.

Parameters	Con	SV1	SV2	SV3	*p*-value
1st day of lactation					
MFR, %	5.33±1.09^b^	6.87±0.86^ab^	8.26±0.56^a^	8.29±0.48^a^	0.0443
MLR, %	2.13±0.16^d^	2.98±0.23^c^	3.68±0.21^b^	4.01±0.29^a^	0.0301
MPC, %	11.26±1.23^c^	13.95±0.86^b^	15.86±0.59^a^	15.87±0.64^a^	0.0298
SNF, %	14.33±1.26	14.62±2.36	14.23±3.25	14.63±3.38	0.5615
21st day of lactation					
MFR, %	4.16±1.23^b^	5.96±1.02^ab^	7.63±0.96^a^	7.66±0.84^a^	0.0326
MLR, %	2.04±0.15^c^	2.56±0.12^b^	3.00±0.22^a^	3.02±0.32^a^	0.0256
MPC, %	10.26±1.23^b^	11.32±1.98^ab^	13.35±0.62^a^	13.95±1.26^a^	0.0493
SNF, %	10.27±2.36	10.32±2.31	10.30±3.34	10.29±3.21	0.6235

Values with different small letter superscripts in the same column mean a significant difference (*p* < 0.05). MFR, milk fat ratio; MLR, milk lactose ratio; MPC, milk protein content; SNF, solids-not-fat.

### 3.6. Semen vaccariae extracts improved the milk immunoglobulin composition of lactating sows

The IgM, IgA, and IgG contents were 19.00, 19.18 and 19.10% vs. 64.02, 126.78 and 159.83% vs. 9.38, 19.78 and 22.92%, respectively, higher (*p* < 0.05) in SV1, SV2, and SV3 than those in the control group, and the IgG contents were increased (*p* < 0.05) by 9.52% vs. 12.38%, respectively, in SV2 and SV3 compared with SV1, the IgA contents in SV3 were 58.42% vs. 14.58%, respectively, higher (*p* < 0.05) than those in SV1 and SV2 on the first day of lactation. There were no significant differences (*p* > 0.05) in the IgM, IgA, and IgG contents among groups on the 21st day of lactation (Table 8).

**Table 8:** Effect of semen vaccariae extracts on the milk immunoglobulin of lactating sows.

Parameters	Con	SV1	SV2	SV3	*p*-value
1st day of lactation					
IgM, mg/mL	38.06±3.52^b^	45.29±2.09^a^	45.36±2.34^a^	45.33±2.62^a^	0.0459
IgA, mg/mL	2.39±0.54^d^	3.92±0.26^c^	5.42±0.33^b^	6.21±0.34^a^	0.0302
IgG, mg/mL	192±10.21^c^	210±5.63^b^	230±12.12^a^	236±10.36^a^	0.0289
21st day of lactation					
IgM, mg/mL	50.26±3.26	51.33±4.46	51.69±4.68	52.22±4.65	0.5628
IgA, mg/mL	4.01±0.65	4.09±0.98	4.12±0.62	4.23±0.78	0.6258
IgG, mg/mL	240±10.69	239±11.26	241±12.05	242±13.56	0.5495

Values with different small letter superscripts in the same column mean a significant difference (*p* < 0.05). IgA, immunoglobulin A; IgG, immunoglobulin G; IgM, immunoglobulin M.

### 3.7. Semen vaccariae extracts improved the relative gene expression related to mammogenesis of lactating sows

**Figure 1:**
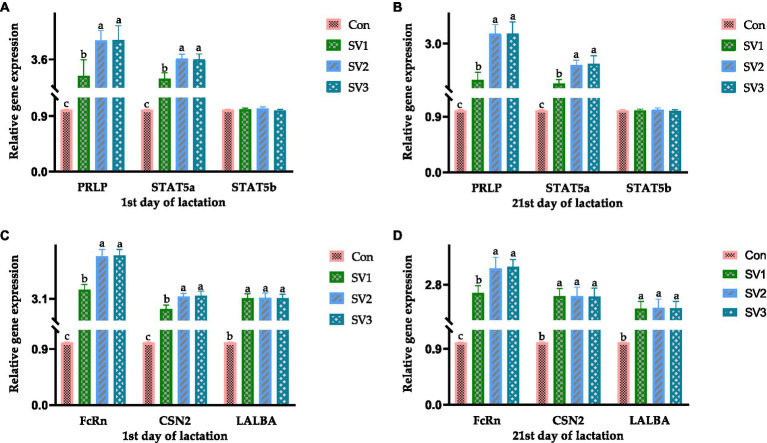
The relative gene expression related to mammogenesis on the 1st and 21st day of lactation. The data of relative gene expression in the four Con, SV1, SV2, and SV3 groups, **(A)** on the first day of lactation, *PRLP* (*Prolactin receptor*): 1.00 ± 0.01^c^, 3.02 ± 0.56^b^, 4.26 ± 0.35^a^, 4.27 ± 0.51^a^, respectively, *p* = 0.0324; *STAT5a* (*Signal transduction and activator of transcription 5a*): 1.00 ± 0.01^c^, 2.92 ± 0.21^b^, 3.62 ± 0.16^a^, 3.60 ± 0.19^a^, respectively, *p* = 0.0223; *STAT5b* (*Signal transduction and activator of transcription 5b*): 1.00 ± 0.01, 1.01 ± 0.02, 1.02 ± 0.03, 0.99 ± 0.02, respectively, *p* = 0.0827; **(B)** on the 21st day of lactation, *PRLP* (*Prolactin receptor*): 1.00 ± 0.01^c^, 2.02 ± 0.21^b^, 3.26 ± 0.25^a^, 3.27 ± 0.31^a^, respectively, *p* = 0.0434; *STAT5a* (*Signal transduction and activator of transcription 5a*): 1.00 ± 0.01^c^, 1.92 ± 0.11^b^, 2.42 ± 0.13^a^, 2.46 ± 0.21^a^, respectively, *p* = 0.0334; *STAT5b* (*Signal transduction and activator of transcription 5b*): 1.00 ± 0.01, 1.00 ± 0.02, 1.01 ± 0.03, 0.99 ± 0.02, respectively, *p* = 0.0738; **(C)** on the first day of lactation, *FcRn* (*Neonatal Fc receptor*): 1.00 ± 0.01^c^, 3.56 ± 0.26^b^, 5.26 ± 0.34^a^, 5.30 ± 0.31^a^, respectively, *p* = 0.0264; *CNS2* (*β-Casein*): 1.00 ± 0.0^c^, 2.57 ± 0.21^b^, 3.21 ± 0.16^a^, 3.25 ± 0.23^a^, respectively, *p* = 0.0261; *LALBA* (*α-Lactalbumin*):1.00 ± 0.01^b^, 3.12 ± 0.23^a^, 3.15 ± 0.24^a^, 3.13 ± 0.20^a^, respectively, *p* = 0.0359; **(D)** on the 21st day of lactation, *FcRn* (*Neonatal Fc receptor*): 1.00 ± 0.01^c^, 2.56 ± 0.21^b^, 3.26 ± 0.31^a^, 3.30 ± 0.21^a^, respectively, *p* = 0.0354; *CNS2* (*β-Casein*): 1.00 ± 0.0^b^, 2.47 ± 0.22^a^, 2.47 ± 0.26^a^, 2.46 ± 0.24^a^, respectively, *p* = 0.0376; *LALBA* (*α-Lactalbumin*): 1.00 ± 0.01^b^, 2.12 ± 0.21^a^, 2.15 ± 0.24^a^, 2.13 ± 0.20^a^, respectively, *p* = 0.0358. Values with different small letter superscripts in the same column mean a significant difference (*p* < 0.05).

The relative *PRLR*, *STAT5a*, *FcRn*, *CSN2*, and *LALBA* expressions in the groups treated with semen vaccariae were 202, 326 and 327% vs. 192, 262 and 260% vs. 256, 426 and 430% vs. 157, 221 and 225% vs. 212, 215 and 213%, respectively, higher (*p* < 0.05) than those in the control group on the first day of lactation. The relative *PRLR*, *STAT5a*, *FcRn*, *CSN2,* and *LALBA* expressions in the groups treated with semen vaccariae were 102, 226 and 227% vs. 92, 142 and 146% vs. 156, 226 and 230% vs. 147, 147 and 146% vs. 112, 115 and 113%, respectively, higher (*p* < 0.05) than those in the control group, and the relative *FcRn* expressions were increased (*p* < 0.05) by 27.34% vs. 28.91%. Respectively, in SV2 and SV3 compared with SV1 on the 21st day of lactation (Figure 1).

## 4. Discussion

The lactation period is an essential phase of the reproductive cycle, mainly because the sows have a high demand for nutrients to support milk synthesis. Furthermore, an adequate supply of milk is essential for the growth and survival of piglets (18). Shi et al. added 1% semen vaccariae extracts to mouse feed and found that it stimulated mammary gland development and enhanced lactation potential in mice (19). Wu et al. found that the soy isoflavones improved sows’ lactation performance (20). These conclusions are consistent with the results of this study that the average daily gain of piglets and total lactation yield in SV2 and SV3 were increased compared with the control group. It may be attributed into the fact that the active substances in semen vaccariae stimulate the mammary gland development of sows, increase their milk production and ultimately improve the daily gain of piglets.

In most mammals, prolactin is essential for maintaining lactation, and prolactin suppresses lactation (17). The neuroendocrine system is the central regulatory unit of the mammary gland. Estrogen and prolactin can promote mammary gland development and initiate and maintain animal lactation (21, 22). It has been shown that PRL regulates the lactation process in animal cells through messenger substances cGMP and cAMP, where cGMP promotes milk synthesis, and cAMP accelerates milk secretion (23). Therefore, estrogen can directly promote the proliferation and differentiation of breast cells, promote prolactin secretion, and induce lactation initiation (24). Many different types of cells in the animal body are equipped with receptors that accept insulin-like growth factor, making insulin-like growth factor a good protagonist in the cell signaling process by targeting tissues to promote cell–cell communication or cell division (25, 26). Insulin-like growth factors are closely related to insulin, carrying the same amount of amino acids as insulin and facilitating the anabolic response of growth hormone (27). Cui Nai Ling, traditional veterinary medicine with seme vaccariae as one of the active ingredients, could upregulate prolactin levels, regulate insulin resistance, and improve the glucose and lipid metabolism disorders in rats (28). In the present study, the insulin-like growth factor-1, insulin, prolactin, and estrogen contents were increased in SV2 and SV3 compared with the control group, consistent with the above results. It may be attributed to the fact that the hormone metabolic levels are regulated after sows absorb different degrees of semen vaccariae. Each hormone content is different in various production stages, and the specific mechanism of the effect needs further study.

Fat, lactose, protein, and solids-not-fat are the main components of milk and essential indicators for milk quality evaluation (29). The main carbohydrate in milk is lactose, which is synthetically converted from glucose in the blood (30). The main component of fat is triglycerides, which can be produced in the mammary gland’s epithelial cells or blood (31). Almost 50% of the energy required by newborn piglets comes from the fat in colostrum, so the higher the fat content, the better the survival and health of the piglets (32). In addition, colostrum contains high immunoglobulin concentration, an essential source of the immune ability of piglets (33). In this study, the colostrum fat ratio, colostrum lactose ratio, and colostrum protein content in SV2 and SV3 were increased compared with the control group, indicating that the semen vaccariae improved the growth performance of piglets by improving the milk quality, which was consistent with the results of average daily gain of piglets and total lactation yield of sows above. Lysine, methionine, threonine, and tryptophan are essential amino acids in pig production (34). Dourmad et al. reported that the optimal standardized digestible threonine-to-lysine ratio in feed benefited multiparous gestating sows (35). Samuel et al. found that diets with increased lysine content are needed in late gestation, and increasing the feed allowance in late gestation is necessary to maintain a positive energy balance throughout gestation (36). Bin et al. found that a diet consisting of 0.48% methionine administered during the late gestation period can improve the production performance of sows and maintain their health (37). In this study, the lysine, methionine, threonine, and tryptophan concentration were increased in SV2 and SV3 in plasma and milk on the 1st and 21st day of lactation, indicating that the semen vaccariae promoted the lysine, methionine, threonine, and tryptophan absorption in the feed and its synthesis of these four amino acids, which echoes the results of the protein content in milk above. Kielland et al. showed that increased immunoglobulin G levels in colostrum will improve the levels of immunoglobulin G in piglets and potentially increase the survival of the piglets (38). Quesnel et al. reported that various ingredients that presumably have immuno-modulating effects (such as plant extracts, prebiotics, and probiotics) increased concentrations of IgG, IgA, and/or IgM in sow colostrum when they were provided during the last weeks of gestation (39). In this study, the immunoglobulin A, G, and M contents were increased in SV2 and SV3 compared with the control group on the first day of lactation, indicating that the semen vaccariae greatly affected the early lactation period of sows. In the late lactation period of sows, it may be due to the changes in the physiological metabolism of sows and the decrease in piglets’ demand for milk, so there is no significant difference in immunoglobulin contents in sows, and there is no substantial change in the immunoglobulin contents in sow milk.

The mammary gland must undergo several changes to form lactating glands, such as epithelial maturation and acinar formation processes, which are primarily hormonally regulated (40, 41). Prolactin is a peptide hormone produced by the pituitary gland and other tissue mammary epithelium, which directly affects mammary gland development by binding to the prolactin receptor in myoepithelial cells and indirectly affects mammary gland development by regulating the progesterone release through the pituitary-ovary axis, and prolactin promotes acinar differentiation in combination with progesterone (42). During lactation, prolactin stimulates the *JAK2* activation by binding to the prolactin receptor via the *JAK*–*STAT* pathway, which phosphorylates *STAT5*, which subsequently binds to the promoter of the milk protein gene and causes it to be transcribed, resulting in milk production (43–45). In this regard, *STAT5a* can synergize with the glucocorticoid receptor to induce milk protein gene expression, and progesterone and glucocorticoids can inhibit apoptosis in MECs (46–48). The *JAK*–*STAT* pathway also induces the members of the SOCS protein family expression, which negatively regulates the prolactin regulatory pathway, thereby co-regulating mammary gland lactation (49). Suckling of milk by young animals also stimulates prolactin release, which induces myoepithelial cell contraction, allowing milk to pass from the acinar into the ducts (50). The *FcRn* gene plays a vital role in the immunoglobulin G transport during colostrum formation and has a secretory role in epithelial cells (51). *CSN2* gene is a member of the beta-casein family; there are two types of casein protein, beta (encoded by this gene) and kappa, which are secreted in animal milk. Beta casein is the principal protein in animal milk and the primary source of essential amino acids for suckling young animals (45, 52). The *LALBA* gene encodes alpha-lactalbumin, a principal protein of milk (53). Alpha-lactalbumin forms the regulatory subunit of the lactose synthase heterodimer, and beta 1,4-galactosyltransferase forms the catalytic component. These proteins enable lactose synthase to produce lactose by transferring galactose moieties to glucose (54). In this study, the relative *PRLR*, *STAT5a*, *FcRn*, *CSN2,* and *LALBA* expressions in the groups treated with semen vaccariae were higher than those in the control group. The results of the relative *PRLR*, *STAT5a*, and *STAT5b* expressions were consistent with the PRL contents in serum on the 1st and 21st day of lactation, indicating that the semen vaccariae can improve the mammogenesis by regulating the gene expressions in the *JAK*–*STAT* signaling pathway. The relative *FcRn* expression results were consistent with the immunoglobulin G contents in colostrum, and the milk protein contents on the 1st and 21st day of lactation were compatible with the relative *CSN2* and *LALBA* expressions, indicating the semen vaccariae can improve the milk quality by regulating the *CSN2* and *LALBA* expression.

## 5. Conclusion

In this study, the optimum dosage was 3.0 g/kg semen vaccariae, which increased the average daily gain of piglets and total lactation yield during the 11–21 days and 1–21 days of lactation, increased the serum insulin-like growth factor-1, insulin, prolactin, and estrogen contents on the 1st and 21st day of lactation, increased the plasma and milk Lysine, Methionine, Threonine, and Tryptophan concentrations on 21st day of lactation, increased the milk fat ratio, milk protein content, relative *PRLR*, *STAT5a*, *FcRn*, *CSN2*, and *LALBA* expressions on the 1st and 21st day of lactation.

## Data availability statement

The original contributions presented in the study are included in the article/supplementary material, further inquiries can be directed to the corresponding authors.

## Ethics statement

The animal studies were approved by all procedures involving care and management were approved by the Institutional Animal Care and Use Committee of the Chinese Academy of Tropical Agricultural Sciences (Approval No. CATAS-20221019-3). The studies were conducted in accordance with the local legislation and institutional requirements. Written informed consent was obtained from the owners for the participation of their animals in this study.

## Author contributions

CX: Writing – original draft. JX: Writing – original draft. FJ: Software, Writing – original draft. WP: Methodology, Writing – original draft. YS: Data curation, Writing – original draft. XD: Writing – review & editing. HW: Writing – review & editing.
